# Supplementary material for: Seminoma and Embryonal Carcinoma Footprints Identified by Analysis of Integrated Genome-Wide Epigenetic and Expression Profiles of Germ Cell Cancer Cell Lines
Source: PLoS One. 2014 Jun 2;9(6):e98330. doi: 10.1371/journal.pone.0098330 (PMC4041891; doi:10.1371/journal.pone.0098330)
Supplement: File S1 — ZIP file containing DMRforPairs output for significant regions. Please start from the html files. (ZIP) [file pone.0098330.s008.zip › figures/12984.pdf]

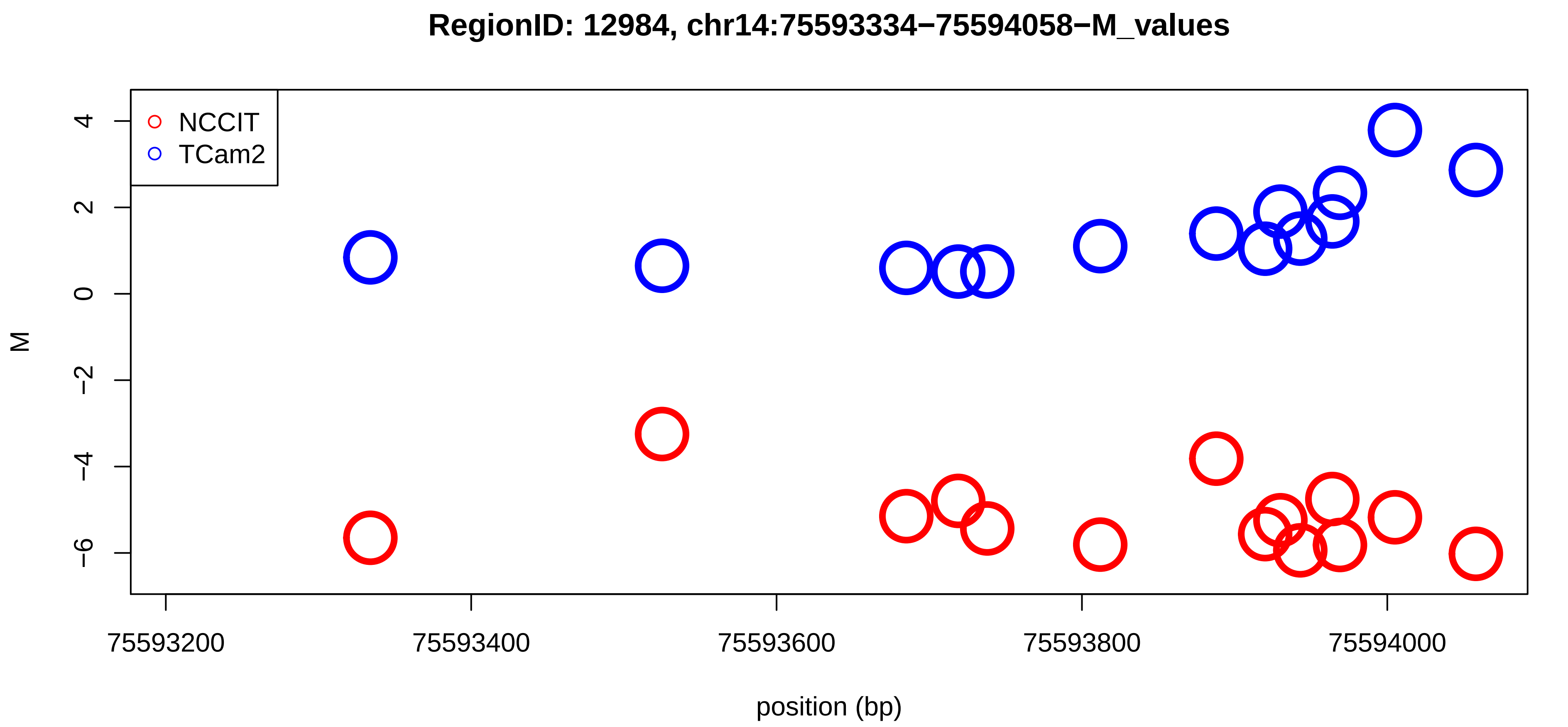
RegionID: 12984, chr14:75593334−75594058−M_values
NCCIT
TCam2
M
4
2
0
−2
−4
−6
75593200
75593400
75593600
75593800
75594000
position (bp)

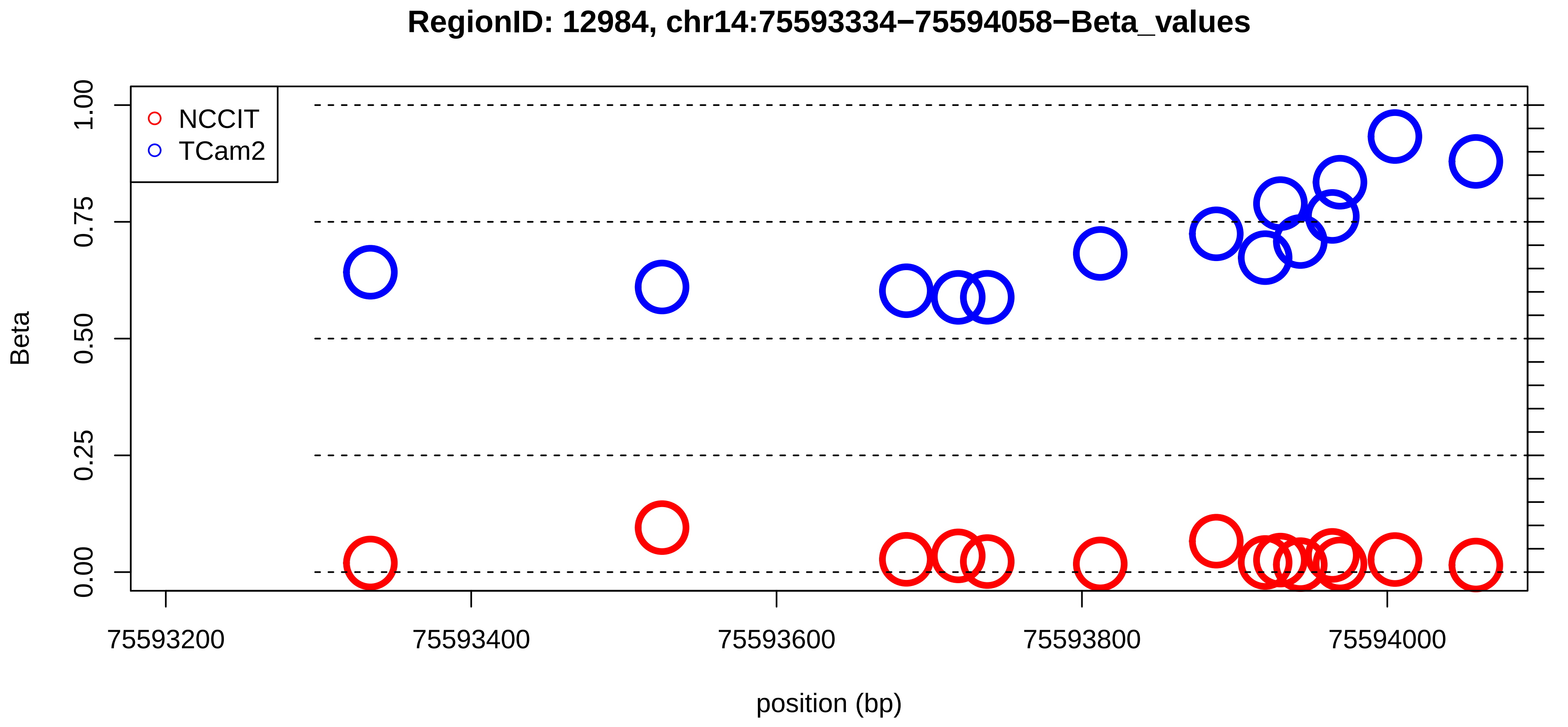
RegionID: 12984, chr14:75593334−75594058−Beta_values
NCCIT
TCam2
Beta
1.00
0.75
0.50
0.25
0.00
75593200
75593400
75593600
75593800
75594000
position (bp)

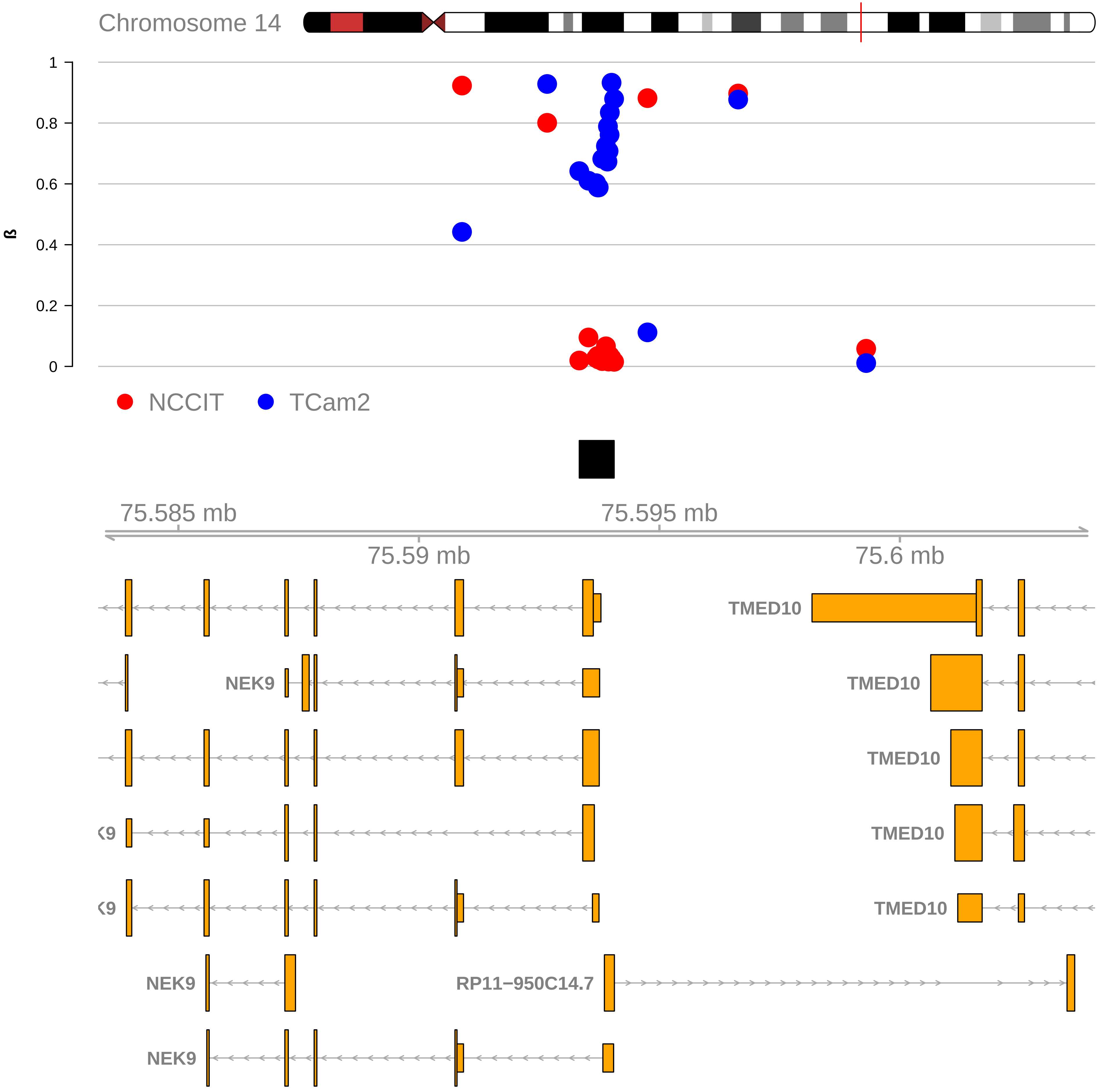

Chromosome 14
1
0.8
0.6
0.4
0.2
0
ß
NCCIT
TCam2
75.585 mb
75.595 mb
75.59 mb
75.6 mb
TMED10
NEK9
TMED10
TMED10
TMED10
TMED10
NEK9
RP11−950C14.7
NEK9
